# Serum cystatin C is associated with the prognosis in acute myocardial infarction patients after coronary revascularization: a systematic review and meta-analysis

**DOI:** 10.1186/s12872-022-02599-5

**Published:** 2022-04-07

**Authors:** Jun Chen, Yang Yang, Chuxing Dai, Yimin Wang, Rui Zeng, Qiang Liu

**Affiliations:** 1 The Third Affiliated Hospital, Zhejiang Chinese Medical University, Hangzhou, 310000 Zhejiang China; 2 The First School of Clinical Medicine, Zhejiang Chinese Medical University, Hangzhou, 310000 Zhejiang China

**Keywords:** Acute myocardial infarction, Serum cystatin C, Prognosis, Coronary revascularization, Meta-analysis

## Abstract

**Objective:**

Our study aimed to assess the association between serum cystatin C levels and prognosis in acute myocardial infarction (AMI) patients after coronary reconstructive surgery.

**Methods:**

We searched PubMed, Embase, and Cochrane Library up to January 21, 2022 without language restriction. Outcomes were major cardiovascular events (MACEs) and mortality. The risk ratio (RR) and 95% confidence interval (CI) were merged by random-effect models.

**Results:**

We included 8 studies with a total of 7,394 subjects in our meta-analysis. Our meta-analysis showed that higher-level of serum cystatin C levels were associated with higher risk of MACEs (RR = 2.52, 95% CI 1.63–3.89, *P* < 0.001) and mortality (RR = 2.64, 95% CI 1.66–4.19, *P* < 0.001) in AMI patients after coronary revascularization. Subgroup analysis showed that the serum cystatin C levels were associated with significantly higher risk of MACEs (RR = 2.72, 95% CI 1.32–5.60, *P* = 0.006) and mortality (RR = 2.98, 95% CI 1.21–7.37, *P* = 0.020) in AMI patients after percutaneous coronary intervention (PCI). However, in AMI patients after coronary artery bypass surgery, there were no significantly higher risk of MACEs (RR = 2.41, 95% CI 0.98–5.93, *P* = 0.05) and mortality (RR = 3.15, 95% CI 0.76–13.03, *P* = 0.10). Further subgroup analysis showed that this significantly higher risk of MACEs and mortality did not change with the study sample size, study population area or study follow-up time.

**Conclusion:**

The meta-analysis demonstrated that higher serum cystatin C levels were associated with significantly higher risk of MACEs and mortality in AMI patients after PCI. It is a biomarker for risk stratification for predicting the prognosis in AMI patients after PCI.

**Supplementary Information:**

The online version contains supplementary material available at 10.1186/s12872-022-02599-5.

## Introduction

Acute myocardial infarction (AMI) is the most severe manifestation of coronary artery disease, which causes over 2.4 million deaths in the USA and over 4 million deaths in Europe and northern Asia, and account for over one-third of deaths in developed countries annually [1]. Ischemic heart disease has become the leading contributor to the burden of the global burden of cardiovascular disease as assessed on the basis of disability-adjusted life-years, and AMI has shifted to low and middle income countries [2]. Coronary revascularization has been regarded as the first-line treatment for AMI patients for decades. Coronary revascularization included percutaneous coronary intervention (PCI) and coronary artery bypass grafting (CABG). Primary PCI could produce higher rates of infarct artery patency and reduce mortality [3].

Cystatin C is a potent cysteine protease inhibitor that plays pleiotropic roles in human vascular pathophysiology, in particular regulating cathepsins S and K [3]. Previous studies suggested that higher serum cystatin C levels were independently associated with an increased risk for future cardiovascular events, even among patients assessed as being at low risk by both estimated glomerular filtration rate (eGFR) values and creatinine levels [4–6]. It is be regarded as a biomarker for cardiovascular disease. However, the association between serum cystatin C levels and prognosis in AMI patients after coronary revascularization is not clear. Several previous retrospective studies reported that higher serum cystatin C levels were also associated with higher risk of major cardiovascular events (MACEs) in AMI patients after coronary revascularization [7–9], but other study showed that significant higher level of serum cystatin C was not an independent risk factor for long-term mortality (HR = 1.08, 95%Cl, 0.34–3.43, *P* = 0.902) [10].

To clarify this question, we conducted a systematic review and meta-analysis to investigate the association between serum cystatin C levels and the prognosis of AMI patients after coronary revascularization.

## Material and methods

This systematic review was conducted in accordance with the Cochrane Collaboration Handbook, observational studies in epidemiology statement [11], Meta-Analysis and Systemic Reviews of Observational Studies (MOOSE) [12], and Preferred Reporting Items for Systematic review and Meta-Analysis (PRISMA) [12]. The Preferred Reporting Items for Systematic review and Meta-Analysis Protocols (PRISMA-P) is shown in Additional file 1.

### Search strategy

**Fig. 1:**
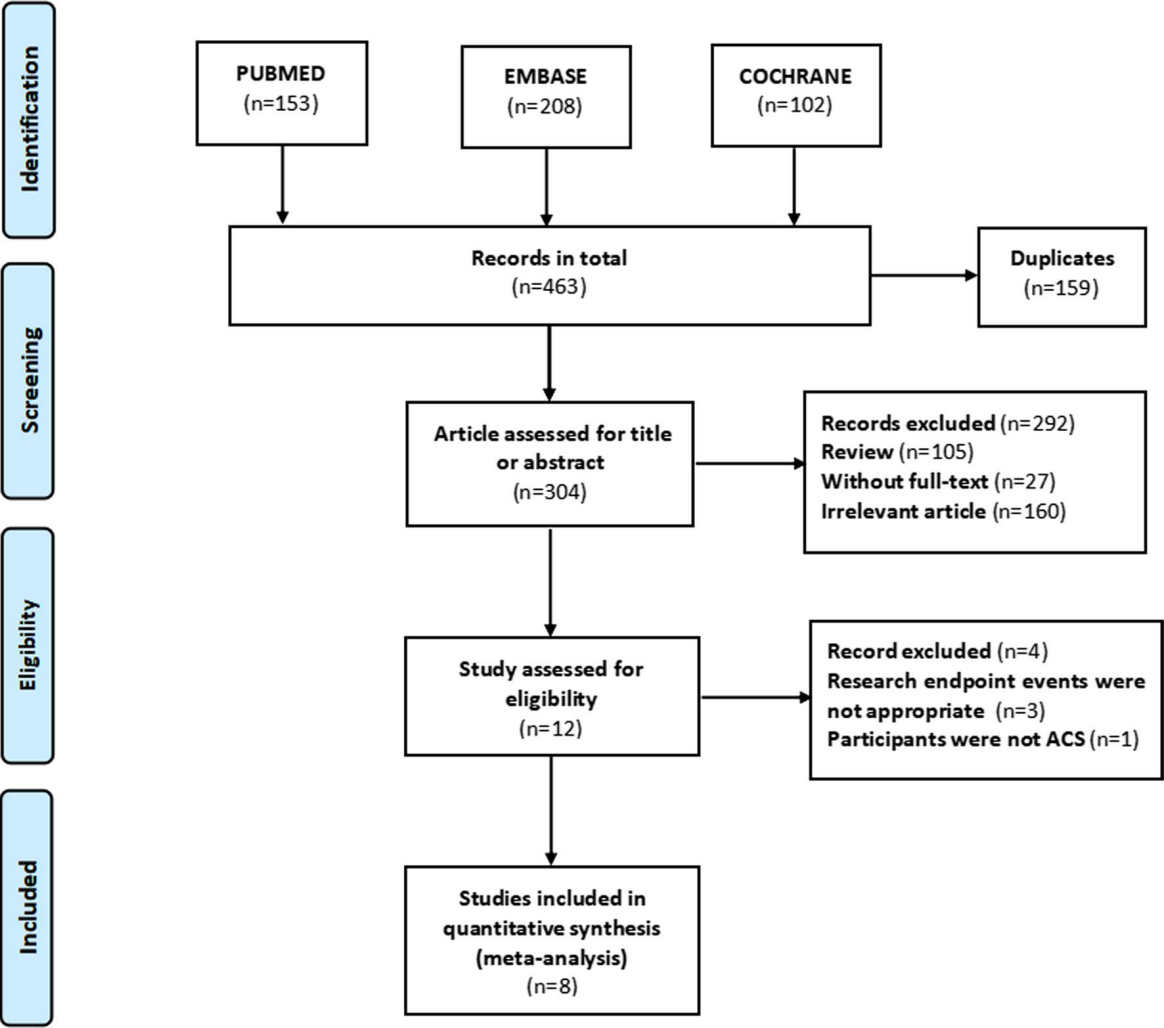
The process of study selection

We searched PubMed, Embase, and the Cochrane Library until January 21, 2022. Search keywords were “cystatin C,” “coronary artery bypass surgery,” and “percutaneous coronary intervention,” without language restriction. Reference lists of related studies were also manually searched and reviewed to identify other potential studies. This study was completed in accordance with the guidelines of Preferred Reporting Items for Systematic Reviews and Meta-Analyses (PRISMA) [12], and the study selection process is shown in Fig. 1.

### Inclusion and exclusion criteria

The included research must meet the following criteria: (1) study included adult patients with AMI who underwent coronary revascularization including PCI or CABG; (2) study reported MACEs and mortality in association with the serum cystatin C level; the MACEs were defined as cardiac death, reinfarction, revascularization, and congestive heart failure; the mortality was defined as all-cause mortality in hospital: any death by fatal cardiac event or other reasons; (3) available data of risk ratio (RR), or hazard ratio (HR) and their 95% confidence intervals (CI) for serum cystatin C level contributed to MACEs or mortality. Exclusion criteria: (1) study with duplicated data, selected the last published article; (2) stable angina pectoris for elective PCI or CABG; (3) conference papers without available data.

### Study selection and data extraction

After excluding duplicates, studies were reviewed to exclude unrelated articles by title and abstract, and then further selected by content. Three reviewers (YY, CXD, and WYM) independently assessed and selected the studies for eligibility. Disagreements were resolved by discussion with the fourth reviewer (LQ). Data were extracted including author, publication year, study type, region, study size, study follow-up, intervention, study outcomes, and adjustment for confounding factors of each study.

### Study quality assessment

The Newcastle Ottawa quality assessment scale (NOS) [13] was used to evaluate the quality of observational studies. The assessment factors included the selection of the study groups, comparability of groups, and ascertainment of the exposure and outcomes. Seven scores or more were considered high-quality studies. The detailed quality assessment is shown in Additional file 2.

### Data synthesis and analysis

We used the risk ratio (RR) and their associated 95% confidence intervals (Cl) to assess the association between serum cystatin C levels and the MACEs and mortality in AMI patients after coronary revascularization, and P value less than 0.05 was considered to be statistically significant. The effect size conduction was merged by a fixed-effect model or random-effect model according to the heterogeneity of the meta-analysis (fixed-effect model for low heterogeneity with *P* > 0.05 or I^2^ < 50%; and random-effect model for high heterogeneity with *P* ≤ 0.05 or I^2^ ≥ 50%). Subgroup analysis analyzed the association between serum cystatin C levels and MACEs according to the intervention (PCI or CABG), ethnicity (Asian vs non-Asian), follow-up duration of study (> 1 year vs ≤ 1 year) and the study sample size (> 500 patients vs ≤ 500 patients). Sensitivity analysis assessed the impact of each study on the results by deleting one study and meta-merging the remaining studies. Sequentially deleting the next study and then meta-merged the remaining ones to test every study’s influence on the final results. If the meta-analysis included more than ten studies, publication bias was assessed by Egger’s test and displayed by a funnel plot. Review Manager version 5.3 (The Nordic Cochrane Centre, The Cochrane Collaboration, Copenhagen) and Stata 14.0 (StataCorp, College Station, TX, USA) were used to conduct our data and perform the meta-analysis.

## Results

### Inclusion of studies and quality assessment

Finally, our study search yielded 463 articles. After removing duplicates, reviews, unrelated studies, and those with unavailable data, eight studies with 7394 subjects were included in this meta-analysis. The The literature selection process is illustrated in Fig. 1. Of these included studies, six studies were prospective observational studies and two studies were retrospective observational studies (Tables 1 and 2). Six studies included the AMI patients receive PCI, and two studies included the AMI patients receive CABG. Five studies were included the Asian population, and three studies were included the European population. Six studies were considered high-quality for their representativeness of the exposed cohort, sufficient follow-up for reliable outcomes, and low risk of bias, while the remaining two studies were considered a medium-quality study for significant clinical heterogeneity (without adjustment for confounding factors) and insufficient follow-up.

**Table 1:** Characteristics of include studies

Author/year	Region	Size	Age (years)	Male (%)	Follow-up	Study type	Intervention	Outcome	Adjustment
Eiji Ichimoto/2009 [28]	Japan	71	64	62 (87.3)	5.6 months	Retrospective	PCI	Cardiovascular events	Age; Killip class; Time from hospital presentation to angioplasty; Prior myocardial infarction; Creatinine;
Doroteia Silva/2012 [7]	Japan	151	61	115 (76.2)	19.2 months	Prospective	PCI	Death/reinfarction	Ejection fraction ≤ 40%;
Tong-Wen Sun/2012 [8]	China	605	60.4	404 (66.8%)	14.3 months	Prospective	PCI	Major adverse cardiac events	NA;
Ozgur Akgul/2013 [9]	Turkey	475	55.8	380 (80%)	1 month	Prospective	PCI	Major adverse cardiac events	Age; gender; diabetes; hypertension; Killip class; anemia; renal failure; No-reflow; Three-vessel disease; unsuccessful procedure; LVEF < 40%;
Fabio Angeli/2015 [29]	Italy	2757	63	2123 (77%)	2 years	Prospective	PCI	All-cause mortality	Age; gender; diabetes; unsuccessful PCI; LVEF; Multivessel disease; Statin prescription at discharge; eGFR;
Seung Hyun Lee/2015 [18]	Korea	1033	65.2	806 (78%)	2.9 years	Prospective	CABG	Mortality and major adverse cerebrovascular and cardiovascular events	NA;
Alain Dardashti/2016 [30]	Sweden	1638	67.4	1295 (79.1%)	3.5 years	Prospective	CABG	All-cause mortality	Age; LVEF < 30%; logarithm of time in ICU; hemoglobin; plasma transfused; creatinine;
Guidong Shen/2018 [31]	China	664	57.6	565 (85.1%)	3.2 years	Retrospective	PCI	Major adverse cardiac events	Age; SBP ≥ 140 (mmHg); NT-proBNP; eGFR; LVEF < 40%;

*PCI* percutaneous coronary intervention, *CABG* coronary artery bypass grafting, *LVEF* left ventricular ejection fraction, *SBP* systolic blood pressure, *eGFR* estimate glomerular filtration rate, *ICU* intensive care unit, *NA* not applicable

**Table 2:** Characteristics of include studies

Author/year	Patients	Intervention	Outcome	Adjustment
Ichimoto/2009 [28]	STEMI	PCI	Cardiovascular events	Age; Killip class; Time from hospital presentation to angioplasty; Prior myocardial infarction; Creatinine;
Silva/2012 [7]	STEMI	PCI	Death/reinfarction	LVEF;
Sun/2012 [8]	AMI	PCI	Major adverse cardiac events	NA;
Akgul/2013 [9]	STEMI	PCI	Major adverse cardiac events	Age; gender; diabetes; hypertension; Killip class; anemia; renal failure; No-reflow; Three-vessel disease; unsuccessful procedure; LVEF;
Angeli/2015 [29]	non-ST-elevation ACS	PCI	All-cause mortality	Age; gender; diabetes; unsuccessful PCI; LVEF; Multivessel disease; Statin prescription at discharge; eGFR;
Lee/2015 [18]	ACS	CABG	Mortality and major adverse cerebrovascular and cardiovascular events	NA;
Dardashti/2016 [30]	CHD	CABG	All-cause mortality	Age; LVEF; logarithm of time in ICU; hemoglobin; plasma transfused; creatinine;
Shen/2018 [31]	STEMI	PCI	Major adverse cardiac events	Age; SBP ≥ 140 (mmHg); NT-proBNP; eGFR; LVEF;

*PCI* percutaneous coronary intervention, *CABG* coronary artery bypass grafting, *LVEF* left ventricular ejection fraction, *SBP* systolic blood pressure, *eGFR* estimate glomerular filtration rate, *ICU* intensive care unit, *m* month, *y* year, *NA* not applicable, *STEMI* ST-elevation myocardial infarction, *ACS* acute coronary syndrome, *CHD* coronary heart disease

### Results of the meta-analysis

**Fig. 2:**
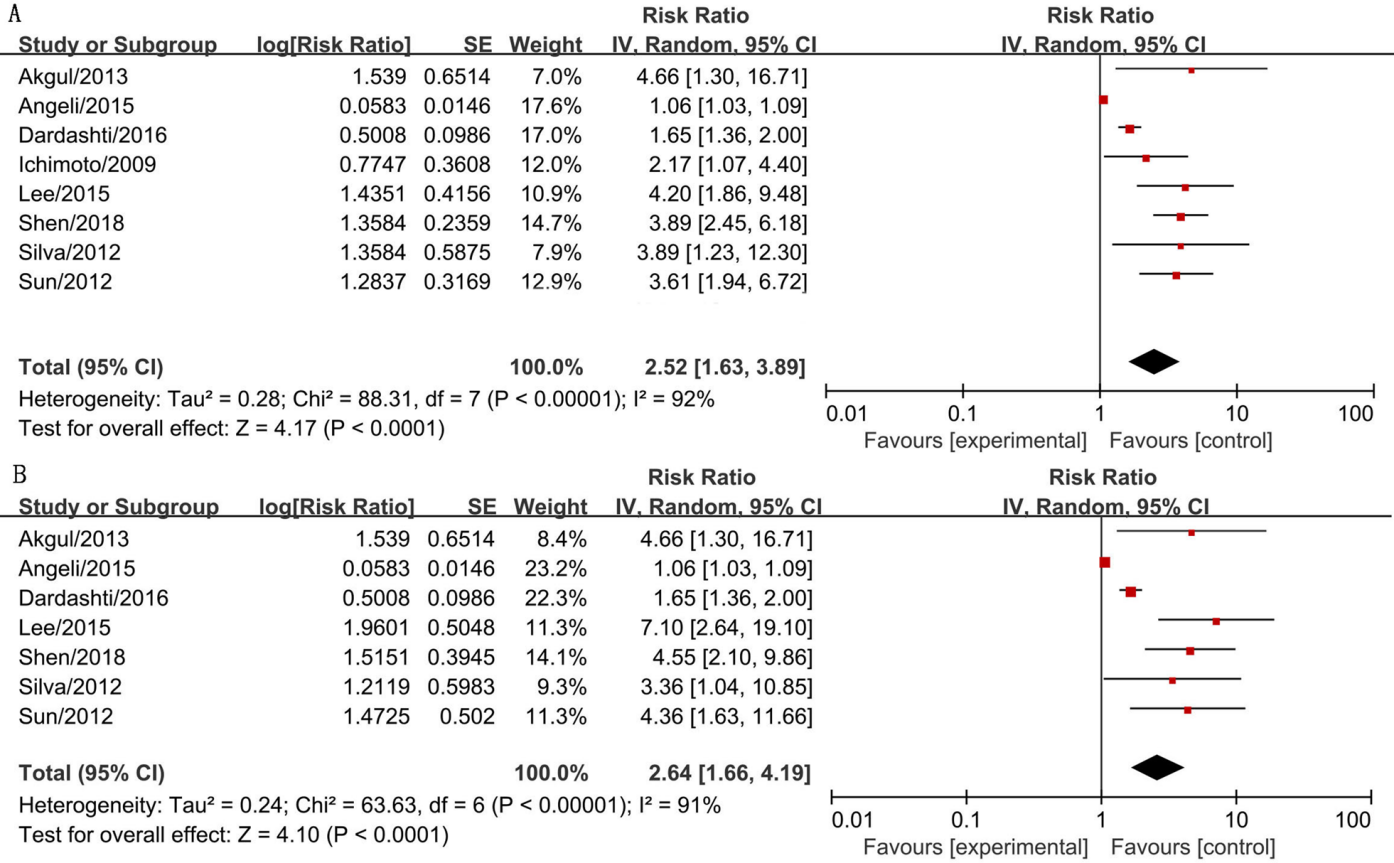
The forest plot of serum cystatin C contributes for the prognosis of AMI patients after coronary revascularization. **a** The association between serum cystatin C and MACE risk in AMI patients after coronary revascularization. **b** The association between serum cystatin C and mortality risk of AMI patients after coronary revascularization. The risk ratio (RR) is used to evaluate the MACE or mortality risk

A total of 7394 subjects (5760 subjects were males) from the included studies were pooled together for the association between serum cystatin C levels and MACEs in AMI patients after coronary revascularization in this meta-analysis. The results showed that high serum cystatin C levels were associated with higher MACEs risk (RR = 2.52, 95% CI 1.63–3.89, *P* < 0.001) and significantly higher mortality risk (RR = 2.64, 95% CI 1.66–4.19, *P* < 0.001) in AMI patients after coronary revascularization with statistic difference (forest plot shown in Fig. 2). These results suggested that serum cystatin C was a predictor for the prognosis of AMI patients after coronary revascularization.

**Fig. 3:**
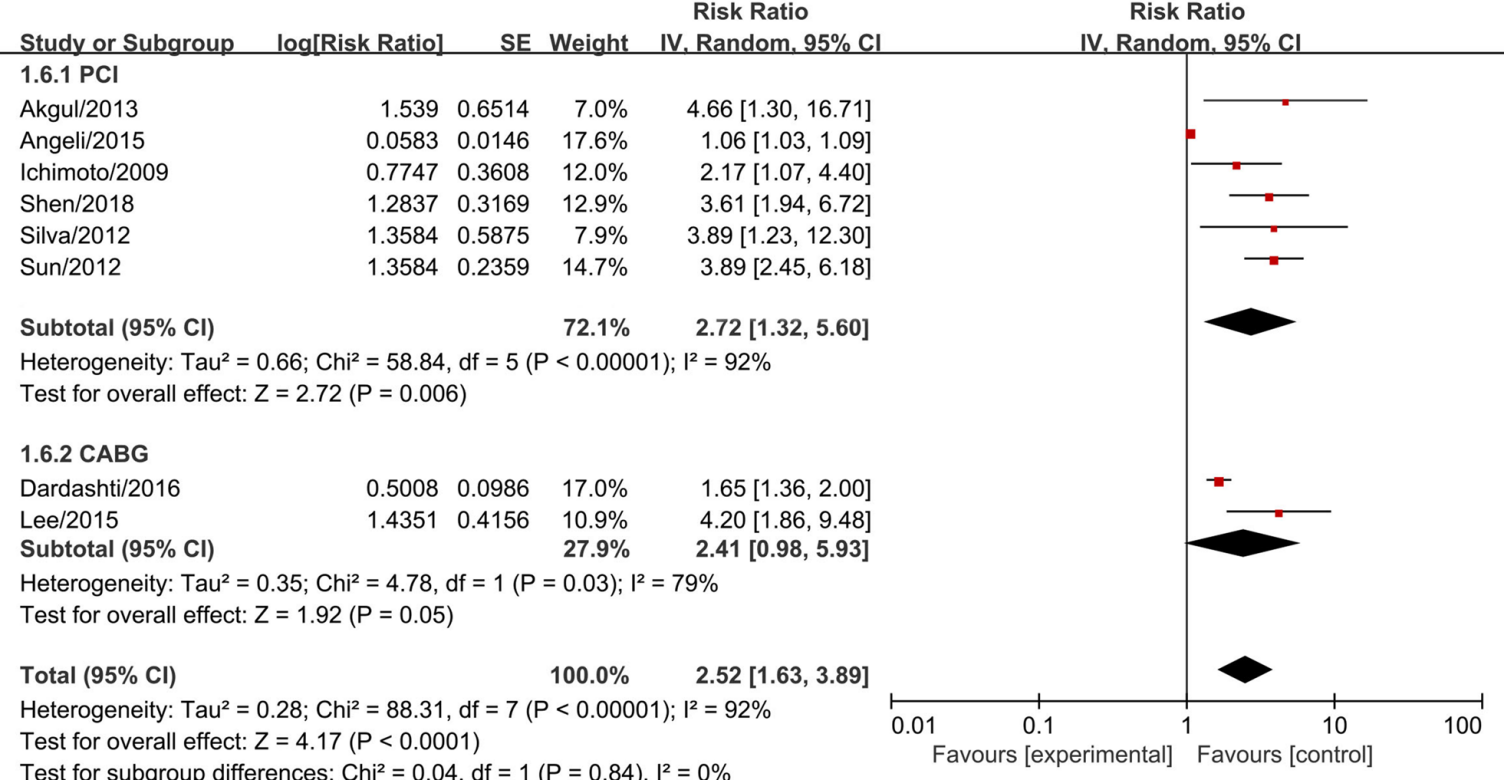
The forest plot of subgroup analysis according to the intervention (PCI or CABG) for serum cystatin C contributes for the MACE risk of AMI patients after coronary revascularization

**Fig. 4:**
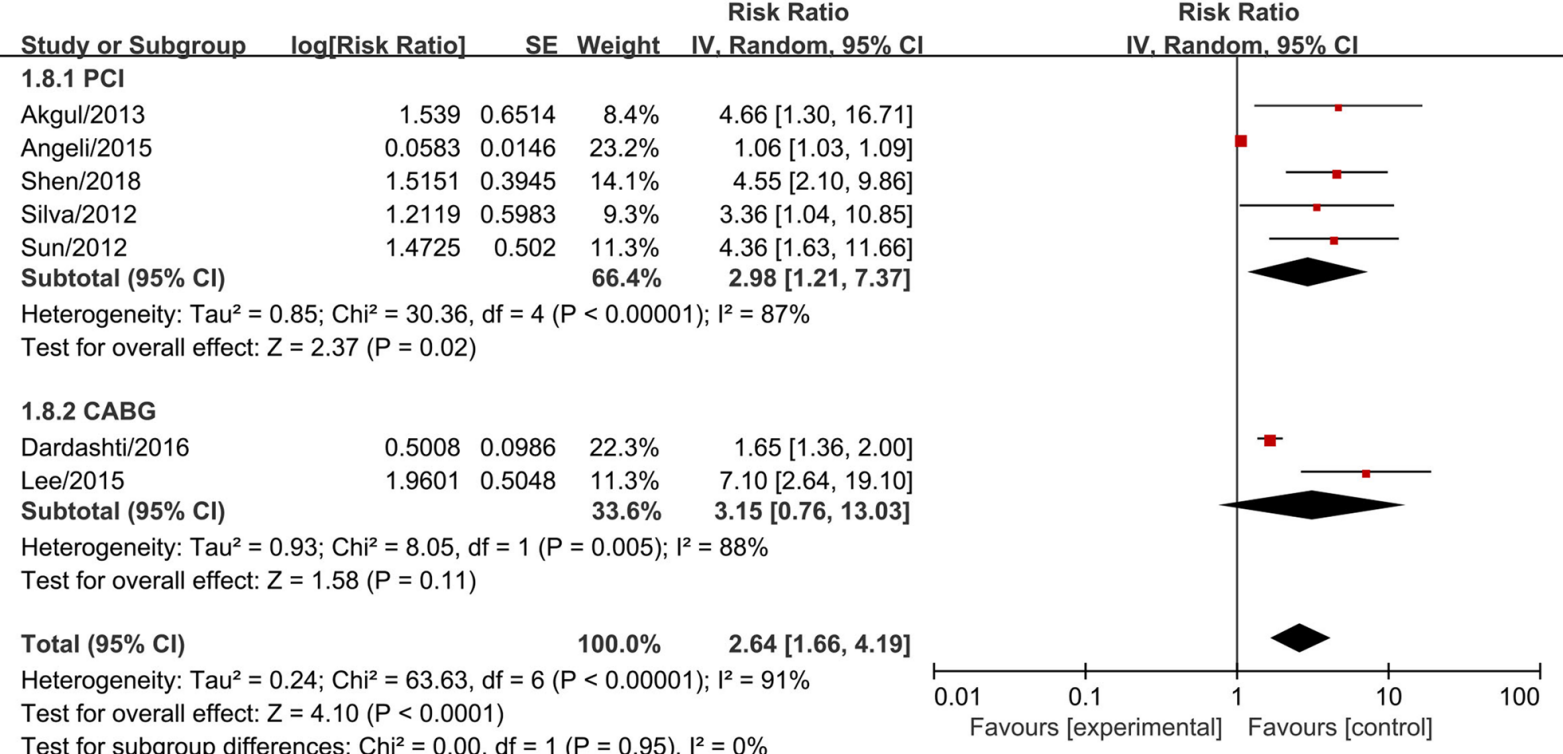
The forest plot of subgroup analysis according to the intervention (PCI or CABG) for serum cystatin C contributes for the mortality risk of AMI patients after coronary revascularization

**Fig. 5:**
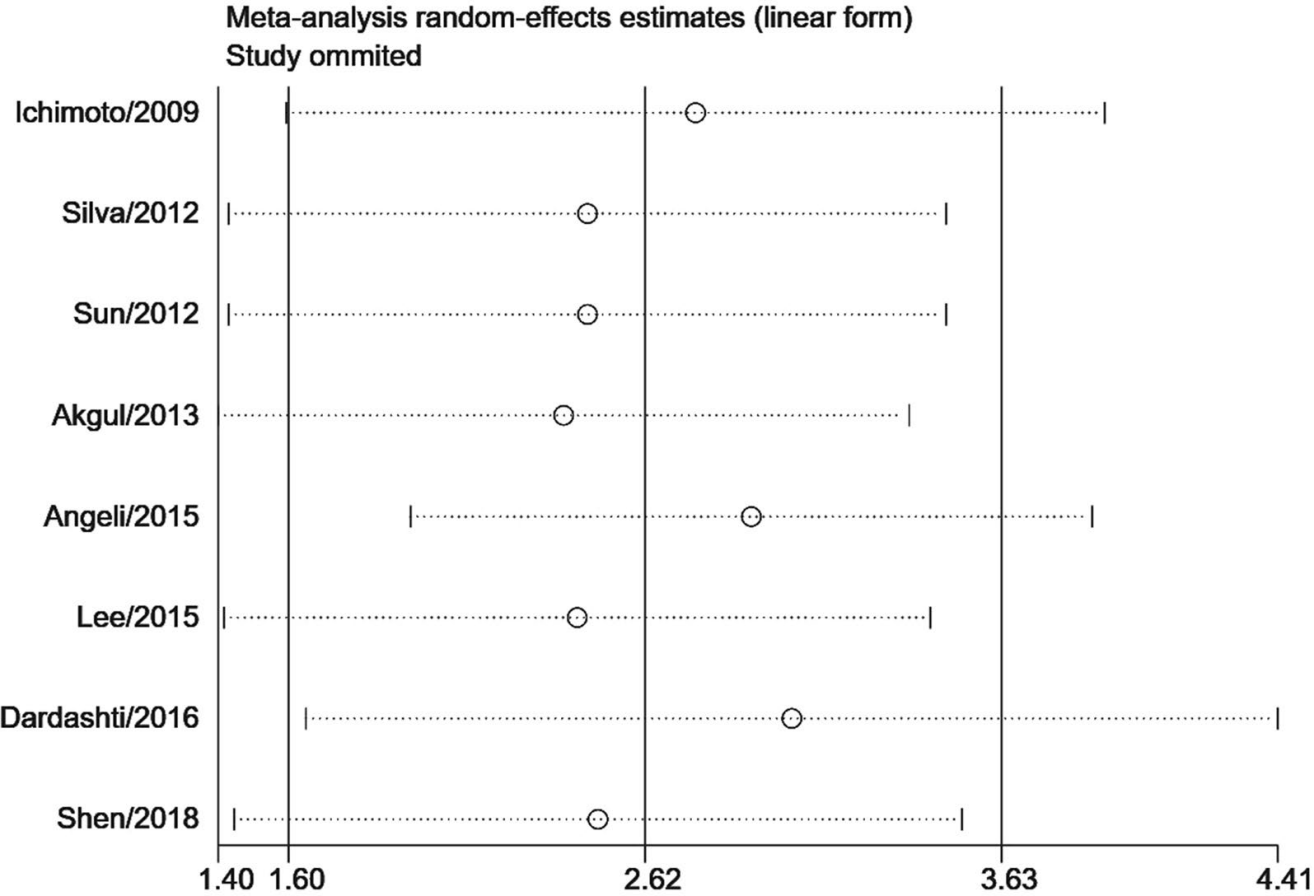
The sensitivity analysis for MACE risk of AMI patients after coronary revascularization. Each branch represents the named study that was omitted; the merged effect size of the studies that remained

**Fig. 6:**
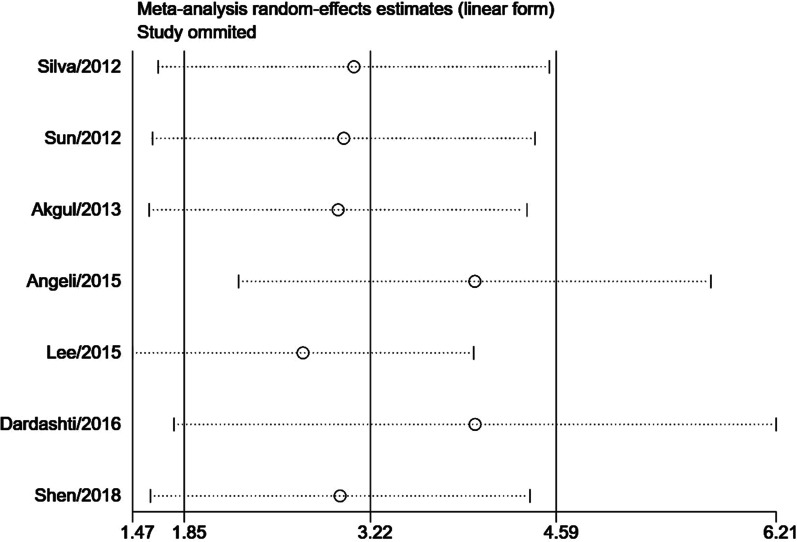
The sensitivity analysis for mortality risk of AMI patients after coronary revascularization. Each branch represents the named study that was omitted; the merged effect size of the studies that remained

Subgroup meta-analysis according to the intervention (PCI or CABG) demonstrated that serum cystatin C was associated with significantly higher MACEs risk (RR = 2.72, 95% CI 1.32–5.60, *P* = 0.006, Fig. 3) and higher mortality risk (RR = 2.98, 95% CI 1.21–7.37, *P* = 0.020, Fig. 4) in studies with AMI patients who received PCI. In AMI patients who received CABG, serum cystatin C was associated with higher risk of MACEs (RR = 2.41, 95% CI 0.98–5.93, *P* = 0.050, Fig. 3) and mortality (RR = 3.15, 95% CI 0.76–13.03, *P* = 0.100, Fig. 4), however, there were no significant differences. Considering the effects of serum cystatin C in different races, we performed a subgroup analysis according to Asian ethnicity vs non-Asian ethnicity. We found that serum cystatin C was significantly associated with higher MACEs risk (RR = 3.49, 95% CI 2.60–4.69, *P* < 0.001) and higher mortality risk (RR = 4.75, 95% CI 2.96–7.63, *P* < 0.001) in the Asian AMI population, while, the non-Asian AMI population did not show a significant association with MACEs and mortality, as shown in Additional file 3: Fig. S1 and Additional file 4: Fig. S2. The significance of this result did not change with the length of follow-up or the size of the study sample, as shown in Additional file 5: Figs. S3, Additional file 6: S4, Additional file 7: S5, Additional file 8: S6. We performed a sensitivity analysis to test the robustness of our results by orderly elimination of each included study and meta-merging the remaining studies. Our results were statistically reliable, as shown in Figs. 5 and 6. Finally, we did not perform a publication bias analysis because fewer than 10 studies were included in this meta-analysis.

## Discussion

Our meta-analysis was based on observational retrospective studies that pooled together 8 studies and 7394 AMI patients after coronary revascularization. To our knowledge, this was the first systematic review to evaluate the association between serum cystatin C levels and the relative MACEs risk of AMI patients after coronary revascularization. The results showed that higher level of serum cystatin C was significantly associated with higher MACEs risk in AMI patients after coronary revascularization. This significant association was independent of study sample size and follow-up time, and was strongly associated with race. Further analysis demonstrated that this significant association mainly in AMI patients after PCI, and based on the current evidence, the significant relationship between higher levels of serum cystatin C and poor prognosis in AMI patients after CABG could not be demonstrated.

Cystatin C is an endogenous cysteine proteinase inhibitor. It is filtered by the renal glomerulus, and metabolized by the proximal tubule which unlike creatinine [14]. It is a sensitive measure of renal function that may be less affected by age, sex, and lean muscle mass than creatinine [15]. Future cardiovascular events are more likely to occur in patients with renal dysfunction than in the general population. Previous studies reported that cystatin C was associated with an increased risk of death and combined events in patients with non-ST elevation acute coronary syndrome, and this association was be regarded for its excellent representativeness of renal function [16, 17]. Simply speaking, the major risk factor for future cardiovascular events is renal dysfunction, and elevated cystatin C levels are only one manifestation of renal dysfunction. In our included studies [9, 18], we also found a significantly higher rate of renal dysfunction in the high serum cystatin C population than in the low serum cystatin C population. However, subsequent studies found that serum cystatin C levels were associated with an increased risk for future cardiovascular events, independent of eGFR values and creatinine levels [4–6]. A recent study reported that elevated cystatin C levels at admission were independently associated with impaired myocardial perfusion, poor cardiac functional recovery and development of congestive heart failure in patients with anterior ST-segment elevation myocardial infarction undergoing primary PCI [19]. These findings suggested that cystatin C may have a specific effect on the cardiovascular system independent of renal function. How cystatin C plays a special role in the cardiovascular system independent of renal function has aroused the interest of cardiovascular researchers.

Acute myocardial infarction (AMI) is always accompanied by a serious pathological basis of coronary artery disorder, except for the myocardial infarction with non-obstructive coronary arteries. The alteration of extracellular matrix composition, calcium deposition and advanced glycation end product-mediated collagen cross-linking aggravate the structural arterial stiffness [20]. Structural arterial stiffness is accompanied by poor vascular dysfunction. The cardio-ankle vascular index (CAVI) is a good tool for evaluating arterial stiffness, which could represent vascular function. It is interesting to note that Yamashita H et al. observed a significant correlation between cystatin C and CAVI [21]. It seems that cystatin C may be related to vascular injury or restoration. Therefore, we hypothesized that cystatin C may be involved in the process of medial destruction of coronary arteries, and may be one of the products. A previous study reported that extracts of atheromatous tissues had approximately twofold greater elastase-specific activity than extracts of uninvolved arteries, mostly due to cysteine proteases [22]. Attenuated atherosclerosis in cathepsin S–deficient mice provided direct evidence for cysteine protease involvement in atherogenesis [23]. Later studies found that inflammatory cells and cytokines associated with atherosclerosis can stimulate the production of lysosomal cathepsins, and increase plasma concentrations of cystatin C [15, 24]. These results suggested that there is a certain dynamic balance between cysteine protease and cysteine statin C in human tissues. Cysteine proteases increase with the aggravation of the activity of atherosclerosis, which induces an increase in cystatin C. The pathological process of atherosclerosis breaks this balance and increases the level of cystatin C, so from this point of view, the level of cystatin C can be used as a biomarker for the progression of atherosclerosis. In addition, Ganda A et al. found that cystatin C levels were positively associated with monocyte counts at baseline after adjusted traditional risk factors [25]. Inflammation and lipid accumulation are two basic hallmarks in the chronic progression of atherosclerosis [26]. Lp(a) was considered an inherited risk factor of atherosclerotic cardiovascular disease, and high serum Lp(a) levels were reported to associated with higher recurrent cardiovascular events risk in AMI patients after PCI [20, 27]. After vascular endothelial injury, the lipid tends to deposit under the intima of the coronary artery, which leads to the infiltration of monocytes, thus lipid and monocytes are the major proinflammatory medium in the disease of atherosclerosis [26]. These results indicated that the high level of cystatin C involves potential mechanisms underlying the strong relationship between monocytes and atherosclerosis. Coronary atherosclerosis is a chronic pathological process that is not terminated after PCI and CABG. Therefore, cystatin C can reflect the continuing coronary artery damage in AMI patients even after PCI and CABG.

In our study, we did not demonstrate a significant association between serum cystatin C levels and the poor prognosis of AMI patients after CABG, which was not consistent with our above hypothesis. However, two included studies about CABG were all showed a significantly higher MACEs risk in the higher serum cystatin C level group. Similar results were observed in the subgroup analysis according to the operation ethnicity (Asian vs non-Asian), Thus, we speculated that it is a statistical dilemma may be caused by limited studies on CABG compared with the studies on PCI. These results would be more accurate and reliable as more CABG studies are conducted in the future.

### Study limitation

Some limitations should be taken into account in our study. First, the sample size was not large enough among each study, and a total of 7394 AMI subjects were included in the present meta-analysis; however, this is the first and largest meta-analysis for investigating the association between serum cystatin C and the prognosis in AMI patients after coronary revascularization. Nevertheless, we did not further analyse the specific classification (STEMI, NSTEMI) of AMI due to the limited number of studies. Considering that there were some different pathological mechanisms between STEMI and NSTEMI, more studies on different types of AMI are necessary in the future. Second, based on the current limited studies, we have not confirmed a significant relationship between cystatin C and poor prognosis in AMI patients receiving CABG; however, we found that two existing studies have shown that high cystatin C levels are significantly associated with poor prognosis in AMI patients receiving CABG. The results will become clearer as more researches are conducted in the future. Third, sex is another potential risk factor in aging adults, given that older females are reported to be at a greater risk for cardiovascular disease than age-matched men. However, based on current studies, we were unable to perform a subgroup analysis based on sex differences to further explore the effect of cystatin C on the prognosis of AMI patients after coronary revascularization in special group (male or female), future research may provide more references based on this topic.

## Conclusion

Our meta-analysis demonstrated that a higher serum cystatin C level was associated with a higher risk of MACEs and mortality in AMI patients after PCI. Serum cystatin C is a biomarker for risk stratification for predicting the prognosis in AMI patients after PCI.

## Supplementary Information

**Additional file 1**. PRISMA-P (Preferred Reporting Items for Systematic Review and Meta-Analysis Protocols) 2015 checklist: recommended items addressed in our systematic review and meta-analysis.

**Additional file 2**. Quality evaluation scale for prevalence studies.

**Additional file 3**.** Fig. S1**: The forest plot of subgroup analysis according to the ethnicity (Asian vs non-Asian) for serum cystatin C contributes for the MACE risk of AMI patients after coronary revascularization.

**Additional file 4**.** Fig. S2**: The forest plot of subgroup analysis according to the ethnicity (Asian vs non-Asian) for serum cystatin C contributes for the mortality risk of AMI patients after coronary revascularization.

**Additional file 5**.** Fig. S3**: The forest plot of subgroup analysis according to the follow-up for serum cystatin C contributes for the MACE risk of AMI patients after coronary revascularization.

**Additional file 6**.** Fig. S4**: The forest plot of subgroup analysis according to the follow-up for serum cystatin C contributes for the mortality risk of AMI patients after coronary revascularization.

**Additional file 7**.** Fig. S5**: The forest plot of subgroup analysis according to the study size for serum cystatin C contributes for the MACE risk of AMI patients after coronary revascularization.

**Additional file 8**.** Fig. S6**: The forest plot of subgroup analysis according to the study size for serum cystatin C contributes for the mortality risk of AMI patients after coronary revascularization.

## Data Availability

The datasets generated and analysed during the current study are available from the corresponding author on reasonable request.

## Abbreviations

AMI: Acute myocardial infarction
PCI: Percutaneous coronary intervention
CABG: Coronary artery bypass grafting
MACEs: Major cardiovascular events
eGFR: Estimated glomerular filtration rate
CI: Confidence intervals
RR: Relative risk
HR: Hazard ratio
LVEF: Left ventricular ejection fraction
