# Supplementary figures and images for: Serum cystatin C is associated with the prognosis in acute myocardial infarction patients after coronary revascularization: a systematic review and meta-analysis
Source: BMC Cardiovasc Disord. 2022 Apr 7;22:156. doi: 10.1186/s12872-022-02599-5 (PMC8991719; doi:10.1186/s12872-022-02599-5)

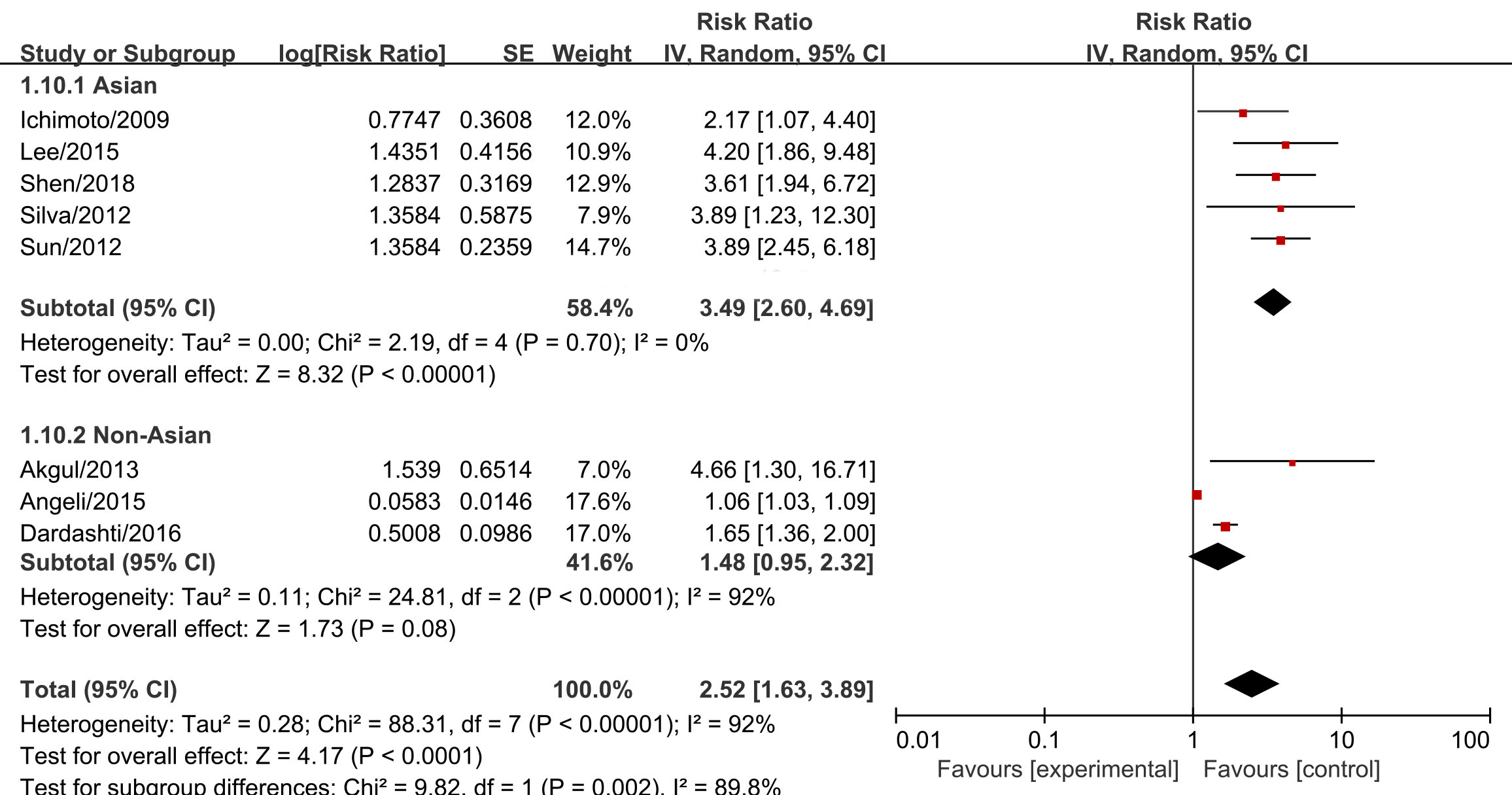

Supplement: Supplementary file 3 — Additional file 3. Fig. S1: The forest plot of subgroup analysis according to the ethnicity (Asian vs non-Asian) for serum cystatin C contributes for the MACE risk of AMI patients after coronary revascularization. [file 12872_2022_2599_MOESM3_ESM.jpg]

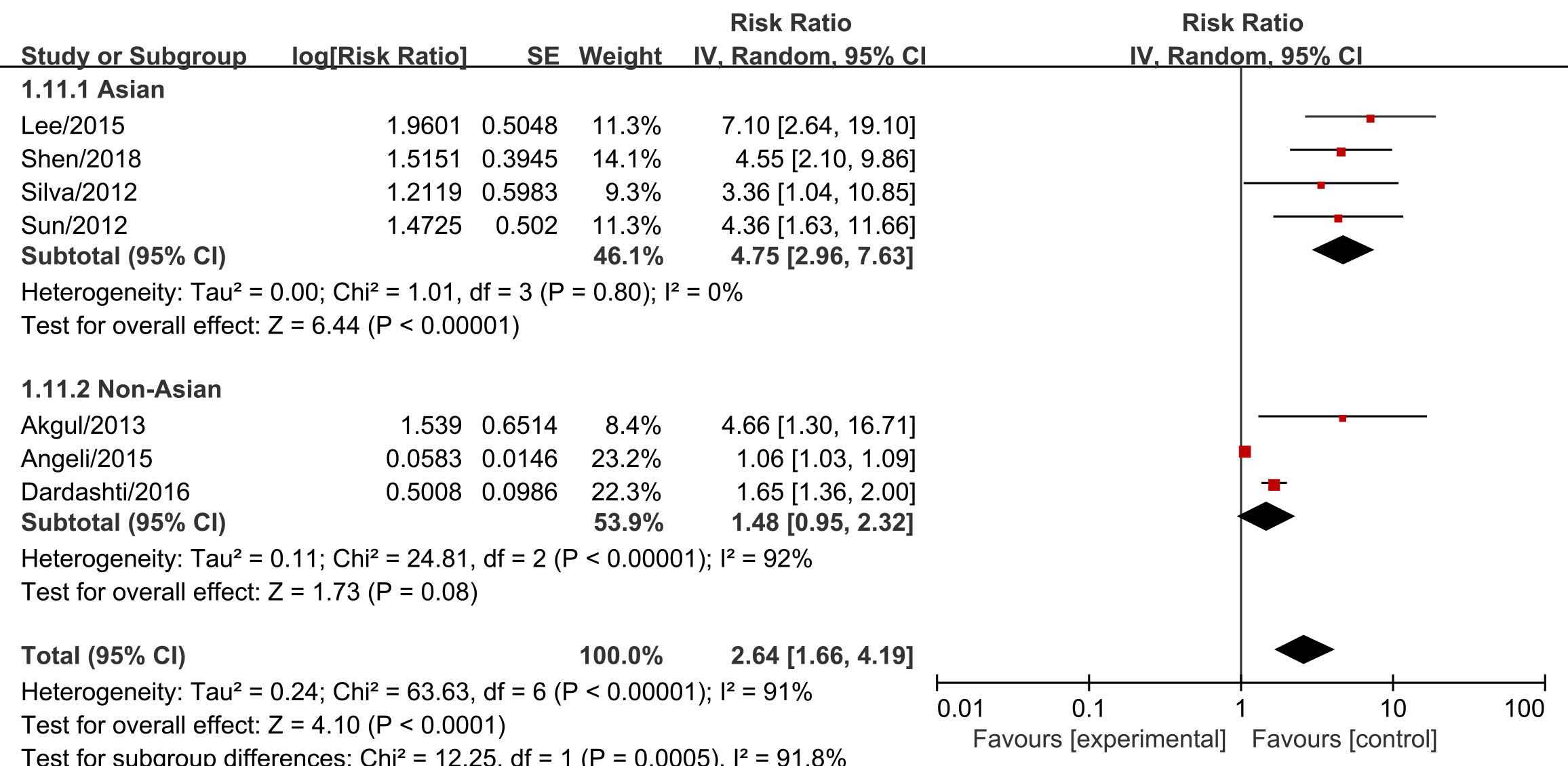

Supplement: Supplementary file 4 — Additional file 4. Fig. S2: The forest plot of subgroup analysis according to the ethnicity (Asian vs non-Asian) for serum cystatin C contributes for the mortality risk of AMI patients after coronary revascularization. [file 12872_2022_2599_MOESM4_ESM.jpg]

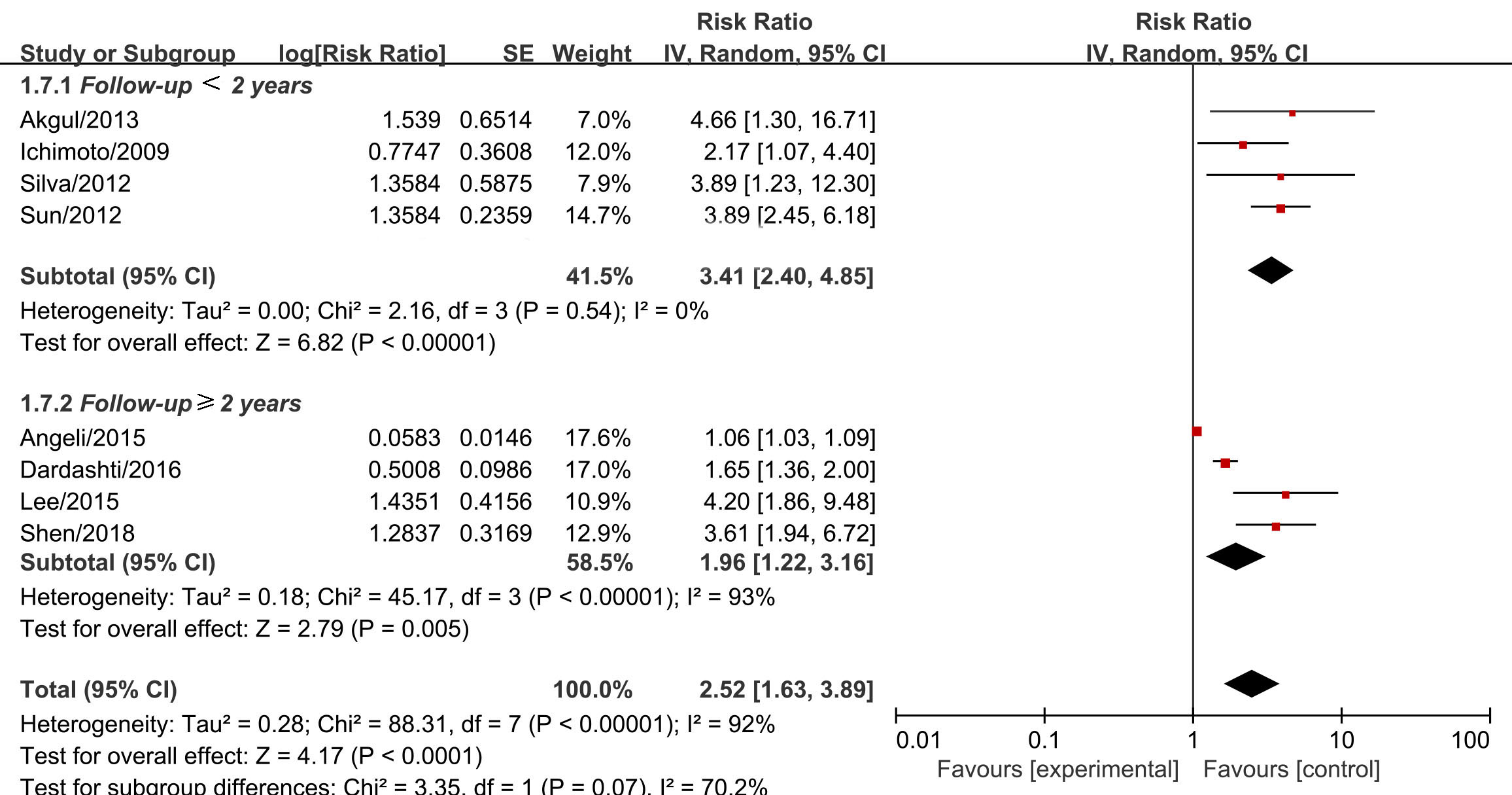

Supplement: Supplementary file 5 — Additional file 5. Fig. S3: The forest plot of subgroup analysis according to the follow-up for serum cystatin C contributes for the MACE risk of AMI patients after coronary revascularization. [file 12872_2022_2599_MOESM5_ESM.jpg]

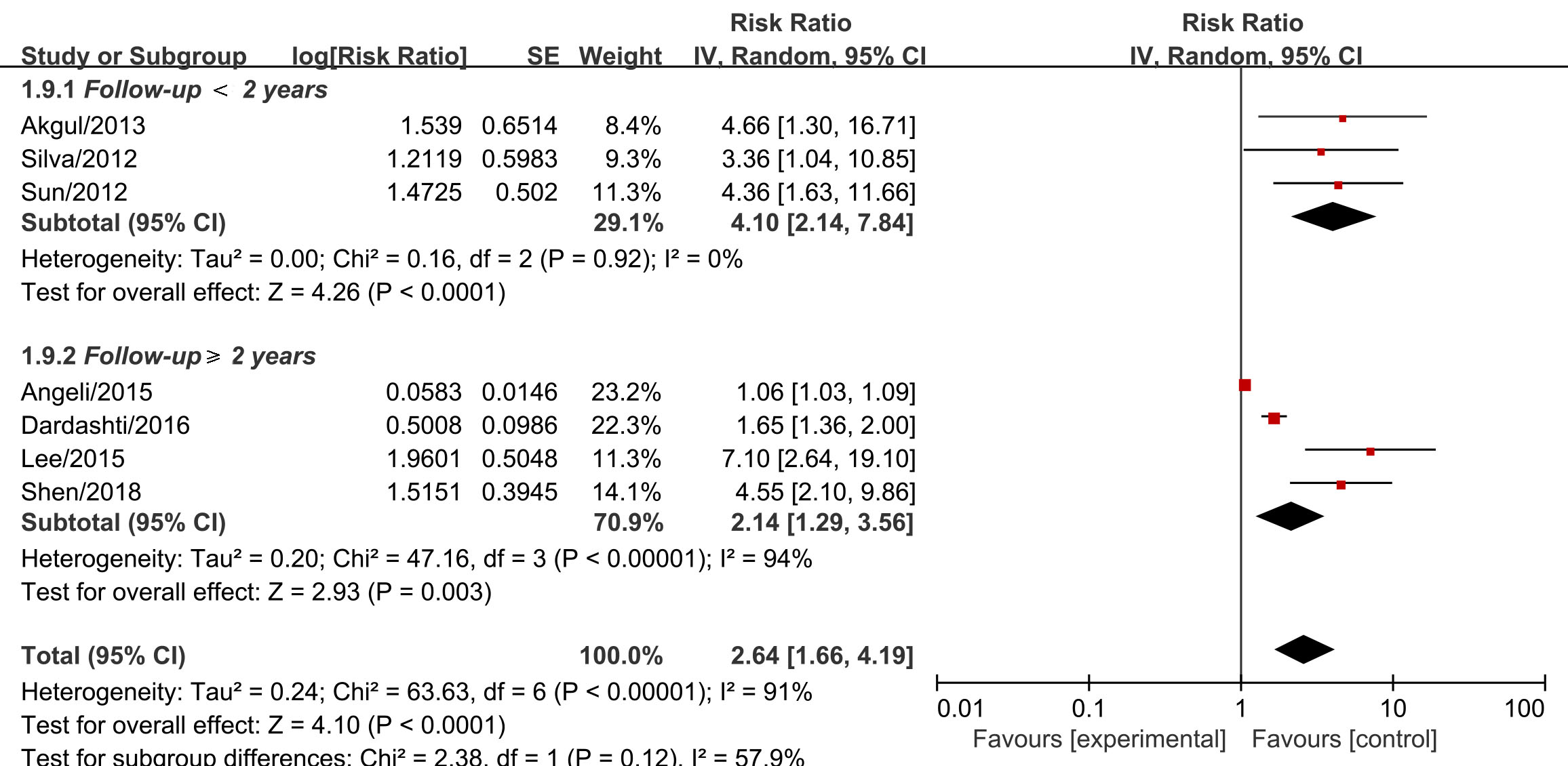

Supplement: Supplementary file 6 — Additional file 6. Fig. S4: The forest plot of subgroup analysis according to the follow-up for serum cystatin C contributes for the mortality risk of AMI patients after coronary revascularization. [file 12872_2022_2599_MOESM6_ESM.jpg]

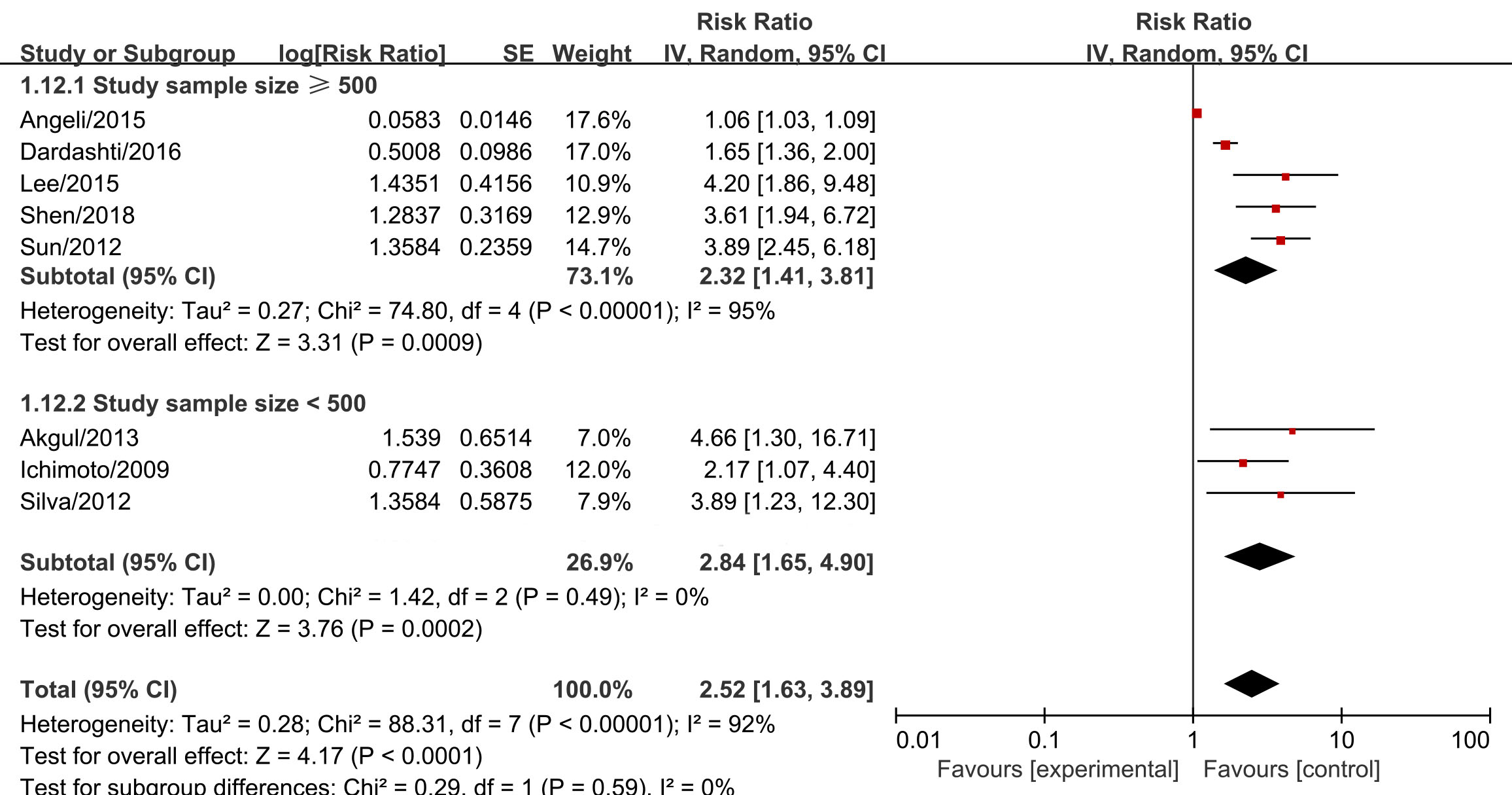

Supplement: Supplementary file 7 — Additional file 7. Fig. S5: The forest plot of subgroup analysis according to the study size for serum cystatin C contributes for the MACE risk of AMI patients after coronary revascularization. [file 12872_2022_2599_MOESM7_ESM.jpg]

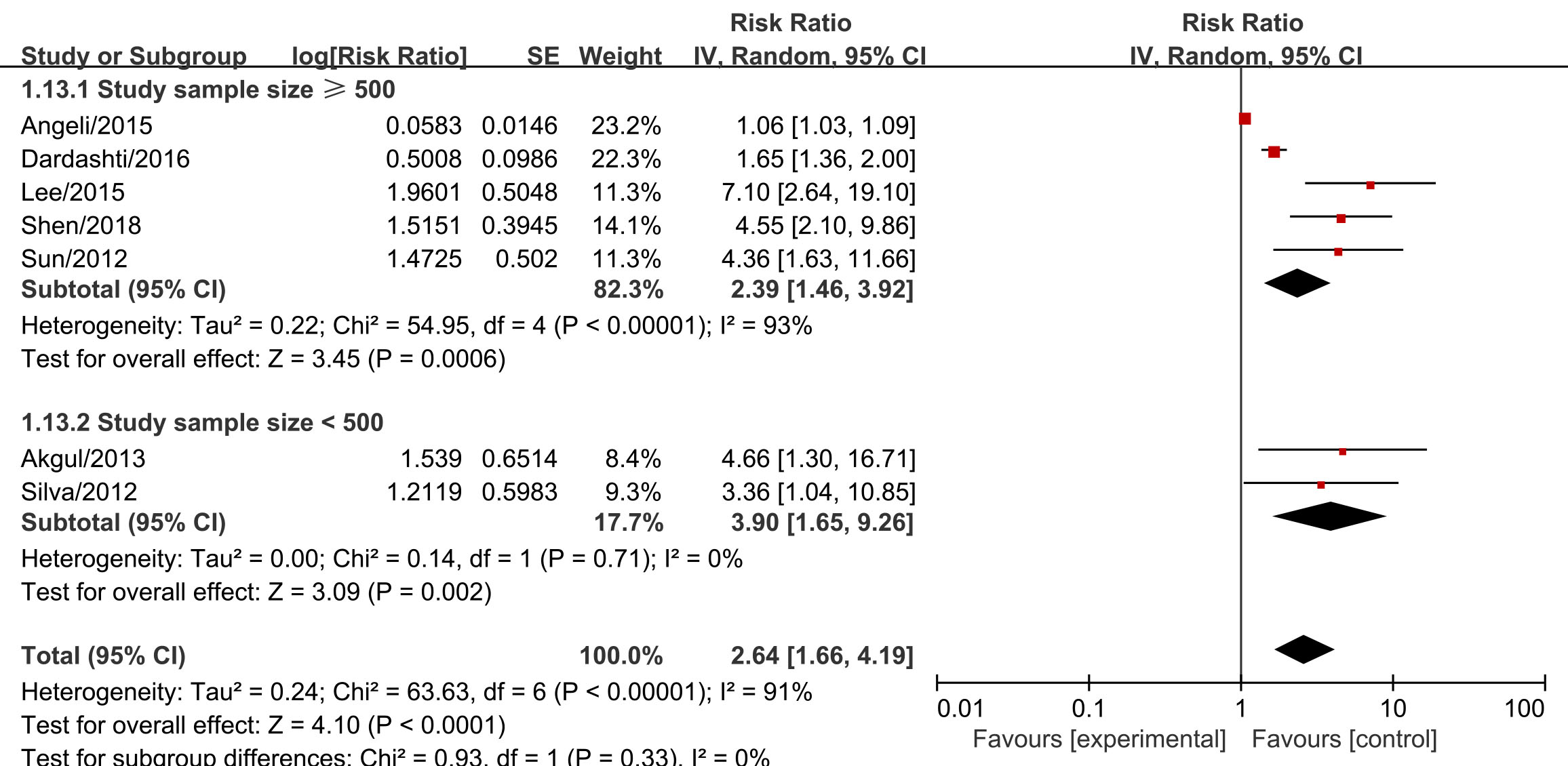

Supplement: Supplementary file 8 — Additional file 8. Fig. S6: The forest plot of subgroup analysis according to the study size for serum cystatin C contributes for the mortality risk of AMI patients after coronary revascularization. [file 12872_2022_2599_MOESM8_ESM.jpg]
